# The Benefits and Detriments of Macrophages/Microglia in Models of Multiple Sclerosis

**DOI:** 10.1155/2013/948976

**Published:** 2013-06-12

**Authors:** Khalil S. Rawji, V. Wee Yong

**Affiliations:** ^1^Hotchkiss Brain Institute, University of Calgary, Calgary, AB, Canada T2N 4N1; ^2^The Departments of Clinical Neurosciences and Oncology, University of Calgary, Calgary, AB, Canada T2N 4N1

## Abstract

The central nervous system (CNS) is immune privileged with access to leukocytes being limited. In several neurological diseases, however, infiltration of immune cells from the periphery into the CNS is largely observed and accounts for the increased representation of macrophages within the CNS. In addition to extensive leukocyte infiltration, the activation of microglia is frequently observed. The functions of activated macrophages/microglia within the CNS are complex. In three animal models of multiple sclerosis (MS), namely, experimental autoimmune encephalomyelitis (EAE) and cuprizone- and lysolecithin-induced demyelination, there have been many reported detrimental roles associated with the involvement of macrophages and microglia. Such detriments include toxicity to neurons and oligodendrocyte precursor cells, release of proteases, release of inflammatory cytokines and free radicals, and recruitment and reactivation of T lymphocytes in the CNS. Many studies, however, have also reported beneficial roles of macrophages/microglia, including axon regenerative roles, assistance in promoting remyelination, clearance of inhibitory myelin debris, and the release of neurotrophic factors. This review will discuss the evidence supporting the detrimental and beneficial aspects of macrophages/microglia in models of MS, provide a discussion of the mechanisms underlying the dichotomous roles, and describe a few therapies in clinical use in MS that impinge on the activity of macrophages/microglia.

## 1. Introduction

The central nervous system (CNS), consisting of the brain and spinal cord, is immune-privileged with access to leukocytes being limited. In several neurological diseases including multiple sclerosis (MS), however, significant infiltration of immune cells from the periphery into the CNS is observed. Demyelination and axonal degeneration are common consequences of CNS inflammation [1]. In addition to extensive accumulation of macrophages, the activation of microglia, the phagocytic cells of the CNS, is a common occurrence following neurological injury [2–6]. This review will discuss the roles of macrophages and microglia as evidenced in the common immune-mediated animal model of MS, experimental autoimmune encephalomyelitis (EAE), as well as in the two prominent demyelinating models of MS, cuprizone and lysolecithin injury.

## 2. Microglia and Macrophages

Microglia and bone marrow-derived macrophages are two genetically distinct myeloid populations [7, 8]. Microglia are the resident immune cells of the CNS and originate from erythromyeloid precursors in the embryonic yolk sac. In early gestation, these precursor cells differentiate into microglia and invade the developing neural tube [7, 9, 10]. In contrast, macrophages are derived from hematopoietic stem cells in the bone marrow. These cells differentiate into blood monocytes which circulate the peripheral vasculature and populate tissues such as the liver, lungs, and nonparenchymal areas of the CNS, including the meninges, choroid plexus, and perivascular space [11, 12]. In the healthy CNS, resting microglia are characterized by many ramified processes, surveying the parenchyma for any possible threats to neurons and macroglia. Under physiological conditions, bone marrow-derived monocytes do not contribute to the local microglia pool [13, 14]. These observations suggest that microglia are sustained by local progenitors. Upon CNS injury, these cells become activated and take on an amoeboid shape, characterized by retracted processes. It is during this state of the CNS when bone marrow-derived macrophages also infiltrate the CNS and accumulate at the injury site, contributing to both further damage and tissue repair [11]. Macrophages within CNS lesion sites are difficult to distinguish from activated microglia, as both are amoeboid-shaped and express many of the same antigenic markers [15]. Due to difficulty in distinguishing these phagocytic cells, many authors refer to these cells collectively as macrophages/microglia.

Although there seems to be a spectrum of different types of macrophages/microglia, there are two main phenotypes that occur prominently in inflammatory lesions. These phenotypes are the classically activated “M1” cells and the alternatively activated “M2” cells [16, 17]. The following discussion is a simplified description as a more sophisticated discussion of these different subsets is beyond the scope of this review (refer to [2, 18]). The M1 macrophages/microglia are generally considered proinflammatory, as they are associated with the secretion of many proinflammatory cytokines including interleukin-1*β* (IL-1*β*) and tumor necrosis factor-*α* (TNF-*α*). M1 cells also express the cell surface markers CD86 and CD16/32 and have inducible nitric oxide synthase (iNOS) activity [11]. The M2 macrophages/microglia on the other hand are anti-inflammatory in nature, as they are associated with the secretion of anti-inflammatory cytokines, such as IL-10. This subclass of macrophages/microglia can be identified by the expression of the mannose receptor (CD206) as well as the enzyme arginase 1 (Arg1). Most cells in the injured spinal cord were found to be of the M1 subset particularly at early stages of injury [19].

## 3. Roles of Macrophages/Microglia in CNS Injury

In the CNS of patients with Alzheimer's disease and MS patients, as well as in their models, a substantial infiltration of macrophages as well as an activation of resident microglia is prominent. This infiltration has been associated with disease severity and represents the predominant immune cell type present in MS autopsy studies [20, 21]. In addition, targeted depletion of CD95 ligand (CD95L), a protein thought to be important in the survival and migration of these cells into the injured CNS, reduced the representation of macrophages in an experimental rodent spinal cord injury model, correlating with enhanced locomotor recovery [22]. Furthermore, treatment with minocycline, an antibiotic that has been thought to reduce the activation of macrophages/microglia and the expression of inflammatory cytokines and matrix metalloproteinases (MMPs), reduced the amount of phagocytic cells in the lesion site, leading to a greater neurological recovery [23–25]. Popovich et al. [26] found that the depletion of macrophages using clodronate liposomes improved neural and motor recovery after experimental spinal cord injury. In addition to these detrimental effects observed *in vivo*, Kigerl et al. [19] showed the ability of M1-polarized macrophages to induce neuronal death. The mechanisms by which activated microglia kill neurons have been amply summarized [27] and include the elaboration of free radicals, proteases, and glutamate. 

Despite these observations of detrimental roles of macrophages/microglia within the injured CNS, many studies have also reported beneficial roles of these innate immune cells. For instance, transplantation of macrophages into the injured CNS promoted the survival of neurons, functional recovery, and nerve regeneration [28]. In the study reporting enhanced nerve regeneration, activated macrophages in the crushed optic nerve were found to secrete a calcium-binding protein, called oncomodulin, which promoted neuron survival and axonal regeneration [29]. Macrophages/microglia have been demonstrated to produce other prorepair molecules, including brain-derived neurotropic factor (BDNF), IL10, and ferritin [30–33]. The latter molecule has been demonstrated to increase the proliferation and differentiation of oligodendrocyte precursor cells (OPCs). In mice expressing a mutated HSV-1 thymidine kinase (TK) gene controlled by the myeloid-specific CD11b promoter, administration of ganciclovir depletes cells of myeloid origin. In a study employing this technique, Barrette et al. [34] was able to show decreased axonal regeneration, increased myelin debris, and severely compromised locomotor recovery in CD11b-TK mice administered ganciclovir. This study demonstrates the reparative role that macrophages play in nerve injury. In mice deficient of CCR2, a receptor involved in recruiting macrophages to the lesion site, accelerated progression of Alzheimer's-like disease was demonstrated, with increasing cognitive impairments and amyloid deposition becoming evident [35, 36]. Another study depleted microglia and subsequently observed the formation and maintenance of amyloid plaques, suggesting that plaques can form independent of microglia [37]. In another study of Alzheimer's-like disease, CD11b-TK mice were again used to show that macrophages play an important role in clearing amyloid deposits [38]. 

**Figure 1:**
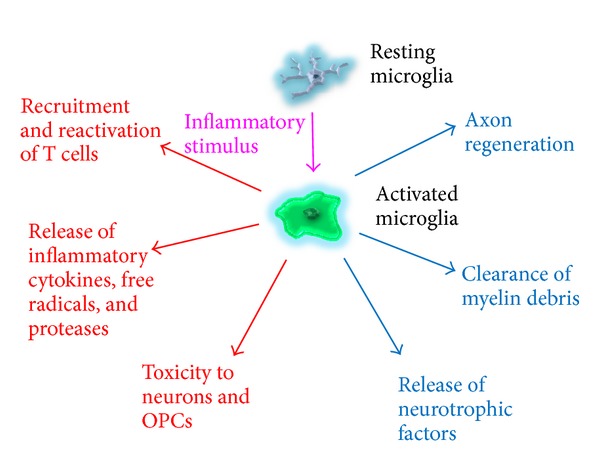
The dichotomy of macrophages/microglia is displayed. Macrophages/microglia have been shown to recruit and reactivate T cells in the CNS and release many detrimental molecules such as proteases, inflammatory cytokines, and free radicals. Through the latter molecules and other mechanisms, macrophages/microglia have been reported to contribute to toxicity to neurons as well as oligodendrocyte precursor cells. Conversely, they have also been observed to aid in axonal regeneration and remyelination as well as assist in the clearance of inhibitory myelin debris. In addition, macrophages/microglia have been shown to release a variety of neurotrophic factors. It can therefore be seen that macrophages/microglia possess an array of detrimental and beneficial functions, with the balance being dictated by the temporal and spatial specifications following CNS injury.

From these studies, it is clear that macrophages/microglia can be both detrimental and beneficial in many different diseases affecting the CNS (Figure 1). To further highlight the dichotomy present in the roles of macrophages/microglia, the remainder of this review will discuss the functions of these cells in three animal models of MS. The reader should bear in mind that although these animal models have yielded a wealth of information concerning the biology of multiple sclerosis, such models represent an artificial mechanism of disease induction.

## 4. MS and Its Inflammatory Model, EAE

MS is a chronic, inflammatory neurodegenerative disease characterized by demyelination and remyelination in the majority of patients in the early phase of the disease. Clinical relapses are thought to be due to multifocal infiltration of immune cells, leading to loss of oligodendrocytes, demyelination, and axonal injury/loss. After a course of the relapsing-remitting part of the disease, most patients enter a phase of the disease characterized by progressive neurodegeneration, manifesting in irreversible disability in MS patients [39]. Demyelinating lesions are often found in the white matter of the brain stem, spinal cord, optic nerve, and cerebellum [39, 40]. Activated microglia and macrophages are frequently observed in active MS plaques [1, 20, 21, 41].

A common animal model of MS is EAE. The disease is induced by immunization with myelin components, including myelin oligodendrocyte glycoprotein (MOG), myelin basic protein (MBP), and proteolipid protein (PLP) [42]. Most studies are carried out with mice immunized with a 35 to 55 residue MOG peptide emulsified in Freund's adjuvant supplemented with *Mycobacterium tuberculosis* extract. Mice may then be injected with pertussis toxin on the day of immunization and then two days later [43]. Along with EAE lesions resembling plaques in MS autopsies, EAE is advantageous in that its myelin-reactive CD4+ T-cell inflammation provides an ample platform for studying the T-cell inflammatory components of MS [43].

Substantial evidence exists for the involvement of macrophages/microglia in EAE. However, as mentioned in other animal models of CNS injury, there is a clear dichotomy in the roles of these phagocytic cells in EAE.

## 5. Detriments and Benefits of Macrophages/Microglia in EAE

The majority of studies demonstrating the detrimental or beneficial involvement of macrophages/microglia in EAE used loss-of-function approaches, in which macrophages/microglia were inhibited or depleted and the subsequent histopathology and behavioural symptoms monitored. For example, in a parabiosis study in which hematopoietic cells were ablated in the recipients but not the donor animals, it was shown that EAE progression was correlated with macrophage infiltration in recipient mice [14]. In other studies, inhibitors to macrophages or their depletion resulted in an attenuation of EAE progression [44–46]. Heppner et al. [47] used CD11b-HSVTK transgenic mice with systemic administration of ganciclovir to reduce macrophage/microglial density. In this study, it was found that ganciclovir administration corresponded to a suppression in the development of EAE, suggesting that macrophage/microglia activation is detrimental in EAE. Agrawal et al. [48] showed that ablation of both matrix metalloproteinase-2 (MMP-2) and MMP-9, which reduced leukocyte penetration across the BBB, suppressed EAE. In addition, Bartholomäus et al. [49] reported the reactivation of CNS-infiltrating pathogenic T cells by MHC class II-presenting macrophages in EAE. In another study, Nikić et al. [50] observed mitochondrial pathology and focal axonal degeneration being initiated by the macrophage-mediated production of reactive oxygen and nitrogen species (ROS and RNS). A regression in axonal degeneration was noticed when this group neutralized the ROS and RNS. In another study providing evidence for a detrimental role of microglia, Rasmussen et al. [51] found that the activation of these cells correlated with degradation of synaptic proteins as well as with atypical phosphorylation of neurofilaments in the cerebral cortex. Another study deleted galectin-1, a critical deactivator of M1 microglia activation secreted by astrocytes, and observed pronounced inflammation-induced neurodegeneration, demonstrating the detrimental effects of M1 polarized microglia [52].

In contrast to these studies reporting detrimental roles of macrophages/microglia in EAE, three studies demonstrated beneficial roles for these cells, particularly those of the M2 variety. In this regard, the transfer of M2-polarized monocytes resulted in a suppression of EAE [53, 54]. Finally, Denney et al. [55] reported that activation of invariant NKT cells increased the proportion of M2 macrophages in the CNS of EAE mice and this led to the attenuation of clinical signs.

In these studies, investigators employed gain-of-function or loss-of-function approaches to determine the roles that macrophages/microglia perform in EAE. When macrophages of the M2 subset were present, EAE severity was dampened, demonstrating a beneficial role for these cells. However, when macrophage activity was depleted, an improvement in EAE resulted, implying a detrimental role for these cells. To further study the role of macrophages/microglia in the processes of demyelination and remyelination, other animal models of MS must be used. In EAE, demyelination and remyelination are difficult to quantitate as the severity, lesion location, and temporal specifications of the pathophysiology vary from animal to animal. Models which can reproducibly induce a focal demyelinating lesion in a white matter tract would offer a significantly better approach to studying the effect that these cells have on the de-/remyelination process. In addition, the latter demyelinating models normally do not have extensive T-cell representation, allowing effects of drugs on macrophages/microglia to be determined. The reproducibility of these models allows for the examination of the effect that loss-of-function, gain-of-function, or pharmacological treatment experiments have on the demyelination/remyelination process [56]. The following sections discuss the roles that have been elucidated for macrophages/microglia in focal demyelinating models, namely, the cuprizone and lysolecithin models.

## 6. The Cuprizone Demyelinating Model

The cuprizone model is the more commonly used of the two main focal demyelinating models, partly due to its ease of administration in the diet to produce injury.

Cuprizone is a copper chelator that is normally fed to mice for 4 to 6 weeks to induce significant, reproducible demyelinating lesions in the corpus callosum, hippocampus, anterior commissure, olfactory bulb, optic chiasm, brain stem, cerebellum, caudate putamen, cerebral cortex, and the cingulum [57–64]. The mechanism of demyelination is thought to be due to selective toxicity to oligodendrocytes, specifically through disruption in the mitochondrial complex IV of these myelin-forming cells. Discontinuation of cuprizone results in remyelination in the corpus callosum, usually through recruitment and differentiation of OPCs [43]. Significant M/M accumulation has been reported in lesions in this model, allowing the specific involvement of these cells in demyelination and remyelination to be examined [59, 61, 65–68]. The cuprizone model is advantageous to other focal demyelinating models in that the insult is easy to administer. However, a longer period is normally required for demyelination to occur, typically taking a few weeks. In addition, once cuprizone is removed, remyelination occurs very quickly, posing a difficulty in examining remyelination-specific events, such as the effect of remyelinating drugs on white matter tracts. 

In the last fifteen years, there has been a large amount of research using this model, yielding many reports of the detrimental and beneficial roles of macrophages/microglia in de-/remyelination. The observed detriments will be first discussed followed by an overview of the beneficial aspects associated with these cells.

## 7. Detriments of Macrophages/Microglia in the Cuprizone Model

Wergeland et al. [69] used the cuprizone model on mice being supplemented with vitamin D3 in their diet. With high and very high doses of this vitamin, the amount of microglia activation was decreased, correlating with a reduction in white matter demyelination. Although these results suggest that activated microglia and infiltrating macrophages may be aiding the demyelination process, one cannot rule out the possibility of vitamin D3 having a protective effect on its own and thereby reducing macrophage/microglia activity. Another study found that administration of erythropoietin to mice fed cuprizone reduced vestibulomotor impairment, an observation that was associated with a decrease in microglia activation in the corpus callosum [70]. Yoshikawa et al. [71] found that pharmacological inhibition of 5-lipoxygenase (5-LO), an enzyme involved in the biosynthesis of leukotrienes, reduced cuprizone-induced axonal damage and motor deficits. Cuprizone-induced demyelination, however, was not attenuated. This observed reduction in axonal damage was associated with a decrease in microglial activation, suggesting that the 5-LO pathway contributes to microglial activation and neurotoxicity. Another study examined the expression of iNOS in the M1 proinflammatory subclass of macrophages/microglia. In that study, the authors found that the administration of fumaric acid esters, compounds which significantly inhibited LPS-induced nitric oxide production by microglia, slightly accelerated remyelination in the corpus callosum [63]. These findings suggest a negative role for iNOS in demyelinating lesions. When cuprizone-treated mice were administered the microglia inhibitor minocycline, a decrease in demyelination and an improvement in motor coordination behaviour was observed [72–74]. 

17 beta-estradiol (E2) is a form of estrogen that has been shown to reduce symptoms in EAE [75]. When this compound was administered to cuprizone-treated male mice, there was a reduction in demyelination accompanied by a delay in microglia activation. This correlation implies a protective effect corresponding to decreased microglia activation. Millet et al. [76] demonstrated that the injection of a proteasome inhibitor, lactacystin, into the corpus callosum during the remyelination process in cuprizone-treated mice resulted in a large improvement in remyelination, corresponding with attenuation of the recruitment of macrophages/microglia. Macrophage inflammatory protein-1*α* (MIP-1*α*) is a protein that has been associated with the recruitment of macrophages/microglia to demyelinating lesions in cuprizone treatment. A study conducted by McMahon et al. [77] found a significant decrease in demyelination in mice deficient in MIP-1*α*, indicating a role of macrophages/microglia in promoting demyelination.

These studies point to a harmful role for macrophages/microglia in cuprizone-induced demyelination. Decreased microglia activation corresponding with decreased demyelination, motor impairment, and increased remyelination, suggests a negative role for microglia in the demyelination/remyelination process. However, there is evidence demonstrating a beneficial role for these cells as well.

## 8. Benefits of Macrophages/Microglia in the Cuprizone Model

In contrast to the studies mentioned above, there are several studies that have noted beneficial roles for macrophages/microglia in cuprizone-induced demyelination. Olah et al. [78] isolated microglia from the corpus callosum of mice in a cuprizone experiment and performed a genome-wide gene expression investigating the upregulated genes associated with remyelination or demyelination. In that study, they found that microglia displayed a phenotype associated with the phagocytosis of myelin debris as well as with the recruitment of OPCs through the expression of cytokines and chemokines. The study provided transcriptomic evidence for the ability of microglia to support remyelination. Jurevics et al. [79] found that genes related to macrophages/microglia appeared in a temporal fashion corresponding to phagocytosis of myelin debris and repair of lesions, suggesting beneficial roles of these cells in clearing the inhibitory environment for repair. Another study looked at the role astrocytes played in providing prorepair signals to microglia in a demyelinated environment. Skripuletz et al. [80] ablated astrocytes in the cuprizone model using a TK transgene under the control of the astrocyte-specific promoter, glial fibrillary acidic protein (GFAP). These mice showed a failure to remove collapsed myelin debris, which was associated with a reduction in microglial activation, suggesting the importance of astrocyte signalling to macrophages/microglia in myelin debris clearance. 

The major histocompatibility complex class II (MHC II) is predominantly present on microglia to present antigens to lymphocytes crossing the BBB. In MHC II null mice, there was delay in remyelination and differentiation of OPCs, pointing to the potential beneficial role played by microglia in remyelination [81]. The same group also found that in mice lacking the microglial enzyme, iNOS, and in mice lacking the microglial cytokine, tumor necrosis factor-*α* (TNF-*α*), there was a significant delay in remyelination and more severe demyelination, indicating the importance of the macrophages/microglial expression of iNOS and TNF-*α* in remyelination [82, 83]. Morell et al. [84] suggested that macrophages/microglia may be important in recruiting OPCs and stimulating their differentiation into mature myelinating oligodendrocytes. This was postulated when they observed that upregulation of mRNA transcripts for myelin-associated glycoprotein (MAG) and myelin basic protein (MBP) coincided with the accumulation of macrophages/microglia, before any remyelination was observed. Mason et al. [85] induced demyelination with cuprizone in mice lacking interleukin-1*β* (IL-1*β*), a cytokine normally secreted by macrophages/microglia. In that study, they found an impairment in remyelination in the homozygous IL-1*β* null mice, pointing to the macrophage/microglial-derived secretion of IL-1*β* as a potentially beneficial molecule for remyelination. 

From these studies, it is evident that the cuprizone model of demyelination has been able to provide a large body of experimental evidence concerning the beneficial roles played by macrophages/microglia in de-/remyelination. These studies have demonstrated that macrophages/microglia have the potential to clear myelin debris, promote the recruitment and differentiation of OPCs, and release cytokines which may be beneficial for the remyelination process. As to when these cells are detrimental or beneficial will most likely depend on other mediators in the microenvironment, the extent of injury, and the temporal coordination between signalling molecules from other sources such as astrocytes. The following sections will now examine the body of evidence accumulated in the other commonly used focal demyelination model, the lysolecithin injury.

## 9. The Lysolecithin Demyelinating Model

Lysolecithin (lysophosphatidylcholine) is a demyelinating chemical that is administered through a stereotactic injection into white matter tracts in the CNS [43]. The dorsal and ventral funiculi of the thoracic and lumbar spinal cord are the most common injection targets, although the corpus callosum is sometimes used as well. Noticeable demyelination occurs hours after injection of the chemical, with significant demyelination lasting about seven to ten days. Substantial remyelination is normally evident twenty-one days after lesion formation [86]. Considerable macrophage infiltration and activated microglia are observed in the lesions with minimal T-cell involvement [87, 88]. Imai et al. [88] found that transplanted GFP-positive, bone marrow-derived macrophages represented the major cell population in lysolecithin-induced mice at days 2, 4, and 7-time points that are early in the demyelination/remyelination process. This model is advantageous in that it is quick to induce with a prolonged remyelination period, thus allowing processes promoting or interfering with remyelination to be studied more comprehensively than with the cuprizone model.

## 10. Detriments of Macrophages/Microglia in the Lysolecithin Model

The lysolecithin model has provided considerable insight into the biology of macrophages/microglia in the de-/remyelination process. Although not as frequently used as the cuprizone model, due largely to its greater challenge in producing injury as it involves a stereotaxic surgery, it has significant potential in elucidating the impact of specific treatment and intervention on demyelination and remyelination. In particular, its rapid induction of demyelination as well as its drawn out course in remyelination offers significant advantages over the cuprizone model, characteristics which may draw in more researchers into employing this model in the study of remyelination.

The detriment of macrophages/microglia in the lysolecithin model was suggested by the result that corticosteroid-treated animals had an enhancement in remyelination associated with a reduction in the number of macrophages/microglia at the lesion site [89]. Another study administered progesterone to adult male mice with lysolecithin demyelination [90] and found that on day 7, there was reduced representation of macrophages/microglia whilst remyelination, and numbers of OPCs and oligodendrocytes were elevated. Furthermore, Schonberg et al. [91] activated macrophages/microglia using zymosan, a toll-like receptor-2 agonist, following lysolecithin injury and observed a loss of OPCs and oligodendrocytes; the results suggest that macrophage/microglia activation can hinder remyelination through an inhibition of OPC recruitment and differentiation.

In summary, there is good data suggesting the negative roles of macrophages/microglia in demyelination/remyelination. As demonstrated in the aforementioned studies, when macrophages and microglia are diminished, the recruitment and differentiation of OPCs are increased, resulting in a better outcome for remyelination. This macrophage/microglia-mediated impairment of OPC recruitment and differentiation may be due to a variety of factors, including the release of toxic molecules. The next section will outline evidence for the positive roles of these cells in lysolecithin-induced demyelination. 

## 11. Benefits of Macrophages/Microglia in the Lysolecithin Model

The benefits of macrophages/microglia in the lysolecithin model was first highlighted by Triarhou and Herndon [92, 93] who showed that depleting macrophages with silica quartz dust and dexamethasone resulted in an impairment in the clearance of collapsed myelin debris as well as a delay in the remyelination process. Using the clodronate liposome method to deplete macrophages, Kotter et al. [94] described that the depletion of macrophages soon after injury (day 1) significantly reduced remyelination 21 days after lysolecithin demyelination. When the authors administered clodronate liposomes later in the remyelination phase (day 8), the outcome of remyelination did not change, indicating that infiltrating macrophages were important in the early stages of remyelination. A later study carried out by the same group [95] found that depletion of macrophages resulted in delayed recruitment of OPCs to the lesion site; the mechanism of the requirement of macrophages was likely related to their clearance of myelin debris that otherwise was an inhibitory milieu for the differentiation of OPCs [96]. A recent study paired the circulatory systems of a GFP-expressing young mouse with a lysolecithin-induced demyelinated old mouse and found macrophages from the young mouse infiltrating the lesion in the old mouse and stimulating an increase in OPC differentiation as well as remyelination [97]. This study very clearly demonstrated the beneficial role macrophages play in promoting remyelination in the lesioned CNS as well as served to demonstrate the ability of young macrophages to enhance the recovery of an aged CNS, an environment in which remyelination and macrophage/microglia accumulation were significantly delayed. In support of the benefits of macrophages/microglia, the early administration of minocycline to demyelinated rodents inhibited macrophage/microglia activation examined at 1 and 3 days postlesion and reduced oligodendrocyte repopulation and remyelination [98]. 

This model of demyelination has accumulated a significant amount of knowledge demonstrating the benefits and detriments of macrophages/microglia in remyelination. While there is data suggesting that these cells may be inhibiting the recruitment of OPCs to the lesion, there is evidence suggesting the alternative. The recurrent observation of these cells being critical in the clearance of inhibitory myelin debris seems to be a requisite for the migration and subsequent differentiation of OPCs into the lesion. Certainly, the temporal coordination of cytokines and signalling molecules, the size and extent of the lesion, and the inhibitory properties of the extracellular environment likely dictate whether these cells will be beneficial or detrimental to the remyelination process. More studies using this model will inevitably yield more information on the complex roles characterizing these cells, thus allowing therapies to be developed which take advantage of the beneficial aspects and suppress the detrimental aspects.

## 12. Basis for Detriments of Macrophages/Microglia

There is a large accumulation of evidence demonstrating the harmful aspects of macrophages/microglia in the demyelinated lesion. Mechanisms underlying these detrimental roles may include the recruitment and reactivation of T cells in the CNS through the release of proteases, the production of proinflammatory cytokines, and the release of reactive oxygen species to induce neurotoxicity and OPC toxicity through excitatory amino acids. This section will discuss some of the evidence supporting these possible mechanisms.

 In order for pathogenic T cells to enter the CNS parenchyma, they must be able to cross the parenchymal basement membrane. This is a laminin-containing basement membrane which lies in direct apposition to the CNS parenchyma and venules within the CNS. For these cells to successfully transmigrate this membrane, proteases such as the MMPs must be present. Macrophages/microglia are a major source of MMPs, and when selective MMPs are blocked, the amelioration of EAE clinical signs is observed [48, 99–101]. These observations suggest that the secretion of MMPs by macrophages/microglia aid in the recruitment of pathogenic T cells to the CNS. 

 Macrophages/microglia also assist in the reactivation of T cells once they enter the CNS parenchyma. In order for a T cell to contribute to CNS pathogenesis, it must be reactivated with antigen presenting cells within the CNS parenchyma. It has been observed that macrophages/microglia upregulate MHC II molecules when they are activated. MHC II molecules are required for antigen presentation to T cells [102]. These observations suggest the capability of activated macrophages/microglia to reactivate primed T cells entering the CNS. It should also be noted that the expression of MHC II has been correlated with the infiltration of T cells as well as with the progression of EAE [103].

 In addition to recruitment and reactivating T cells, macrophages/microglia have also been observed to strip myelin, as well as kill neurons and OPCs [104–106]. One of the mechanisms explaining this macrophage/microglia-mediated toxicity is through the production of cytokines, glutamate, and reactive oxygen species [27]. When macrophages/microglia are activated, they release an array of inflammatory cytokines, such as TNF-*α* and IFN-*γ*. These cytokines induce the release of glutamate. Excessive glutamate stimulation on N-methyl-D-aspartate (NMDA) receptors results in mitochondrial death and ultimately excitotoxic neuronal and oligodendrocyte death [106]. When glutamate release is blocked, EAE progression is attenuated, providing evidence for glutamate excitotoxicity as a plausible mechanism for macrophage/microglia-mediated toxicity [107]. In addition to stimulating the release of glutamate, proinflammatory cytokines and chemokines promote inflammation and antigen presentation, thereby mediating the recruitment and reactivation of T cells to the lesion [108]. Finally, the release of free radicals, such as nitric oxide (NO), has been shown to induce oxidative damage to neurons and oligodendrocyte precursor cells [109].

 It is evident that the array of detriments associated with macrophage/microglial activity in MS and its animal models function through many mechanisms of action. Such modes of action include the expression of proteases to aid in the recruitment of T cells into the CNS, the upregulation of molecules associated with antigen presentation, thereby aiding in the reactivation of pathogenic T cells, and through the release of inflammatory cytokines, free radicals, and glutamate. Developing therapies which target these mechanisms will certainly offer hope in the quest for improving the outcome of remyelination. 

## 13. Basis for Benefits of Macrophages/Microglia

From the array of studies conducted with the different animal models of MS, it is clear that macrophages/microglia, although being shown to be predominantly detrimental, have a variety of beneficial effects. The mechanisms of these beneficial effects are thought to be due to the production of growth factors, the removal of inhibitory debris and toxic products, and through the removal of inhibitory extracellular matrix (ECM) molecules. This section entails a discussion of some of the evidence supporting these plausible mechanisms.

Macrophages/microglia have been reported to secrete many well-known neurotrophic factors, which may help in the recruitment and differentiation of OPCs, as well as in the regeneration of axons. Herx et al. [110] showed that the production of IL-1*β* by microglia regulated the production of ciliary neurotrophic factor (CNTF), an important growth factor for the survival of oligodendrocytes. Studies have also demonstrated the production by macrophages/microglia of other neurotrophic factors, such as nerve growth factor (NGF), brain-derived neurotrophic factor (BDNF), and neurotrophin-3 (NT-3) [111, 112]. As mentioned above, Yin et al. [29] reported that axonal regeneration after optic nerve injury was partially due to oncomodulin, a growth factor secreted by macrophages. Overall, there is evidence to support the claim that macrophages/microglia promote remyelination and axonal regeneration through the secretion of beneficial growth factors. A challenge remains, though, in determining the temporal and spatial characteristics in which these benefits are most likely to occur.

Another beneficial mechanism of macrophages/microglia may be through the removal of inhibitory debris or toxic products. As mentioned previously, Kotter et al. [95] found that macrophages were important for the clearance of myelin debris and in enabling subsequent remyelination to occur. These authors found in a later study that myelin debris acted to inhibit OPC differentiation into mature, myelinating oligodendrocytes [96], thus supporting the ability of macrophages/microglia to clear debris as a beneficial role in remyelination. In another study, the depletion of macrophages with silica dust resulted in a hindered clearance of myelin debris and significantly delayed remyelination [92]. In an Alzheimer's-like disease model, it was found that macrophages recruited to the CNS cleared intracerebral A*β* deposits [38]. Altogether, it can be seen that there is evidence supporting the capability of macrophages/microglia in clearing inhibitory myelin debris as well as toxic products such as A*β* deposits, ultimately associating with a decrease in the severity of the disease.

Finally, our group has shown that macrophages/microglia act beneficially to remove inhibitory extracellular matrix molecules. As mentioned previously, CSPGs are normally deposited around a CNS lesion. We have shown that NG2, a type of proteoglycan, hinders the differentiation of OPCs [113]. It was found that the production of MMP-9 by macrophages/microglia cleared the accumulation of NG2 within a demyelinating lesion, leading to subsequent oligodendrocyte maturation and remyelination. 

Although the mechanisms discussed above serve to explain some of the benefits observed with macrophages/microglia, there are likely other positive mechanisms that aid in the remyelination process. One such mechanism may be through the stimulation of astrocytes to secrete trophic factors as well as to reseal the blood-brain barrier (BBB) [114]. By determining the mechanisms involved in the beneficial aspects of these immune cells, we will be able to better target these endogenous processes with therapies that may stimulate remyelination and axon regeneration. This can already be seen by some of the therapies already in use for the treatment of multiple sclerosis. The next section will discuss some of these therapies and briefly describe the mechanisms in which the macrophage/microglia population is targeted.

## 14. Therapies Involving Macrophage/Microglia Activity

 There are currently seven therapies approved by the US Food and Drug Administration (FDA) for treatment of multiple sclerosis. All of these therapies function by modulating the immune system, with many having broad effects affecting multiple components of both innate and adaptive immunity. While T cells are most commonly targeted by these medications, there are reports describing the involvement of macrophages/microglia in these therapies. This section will briefly describe five of the seven licensed therapies in which significant involvement of macrophages/microglia has been documented. 

Glatiramer acetate (Copaxone) is a first-line therapy that has been shown to reduce the relapse rate and progression of disability in patients with relapsing remitting MS [115]. This compound is a random polypeptide composed of four amino acids designed to mimic myelin basic protein (MBP), a critical component of the myelin sheath [116]. The major mechanism of action is to induce T cells of the T helper type 2 subset (Th2). These cells secrete the anti-inflammatory cytokines interleukin-4 (IL-4), IL-5, IL-6, IL-10, and IL-13 and transforming growth factor-*β* (TGF-*β*). In addition to the induction of Th2 cells, glatiramer acetate has been shown to have an effect of macrophages/microglia, with many studies suggesting a role in promoting an anti-inflammatory M2 phenotype. It was found that glatiramer acetate increased the expression of the anti-inflammatory cytokine IL-10 and reduced the expression of the proinflammatory cytokine tumor necrosis factor-*α* (TNF-*α*) in macrophages/microglia [117–119]. Another study found that this compound inhibited the production of NO, suggesting the polarization away from an M1 phenotype [120]. Finally, this compound has been demonstrated to increase the phagocytic activity of microglia and monocytes, possibly contributing to increased myelin debris clearance [121]. Altogether, it appears as though glatiramer acetate has many effects on the macrophage/microglia population, while at the same time polarizing the T-cell response to one of an anti-inflammatory nature.

Interferon-*β* (Avonex, Betaseron, Extavia, and Rebif) is another first line therapy which has demonstrated success in reducing the rate and severity of relapses, as well in slowing the progression of disability [121]. The main mechanisms of action seem to be in the inhibition of T-cell recruitment and activation, as well as in the modulation of cytokines [116, 122, 123]. Though the reduction in the T-cell response is quite evident, aberrations in the macrophage/microglia response may also contribute. For instance, interferon-*β* has been demonstrated to reduce the proliferation of both macrophages and monocytes as well as reduce the expression of MHC II on these cells [124]. This reduced expression of MHC II may be a reason for the decreased activation of T cells, as the antigen-presentation capabilities of macrophages/microglia would be hindered. Another example of macrophage/microglia involvement is demonstrated in the study by Hall et al. [124], which highlighted the reduction of toxic microglia-derived respiratory bursts as a result of interferon-*β* interaction. As interferon-*β* is a cytokine, many other unknown mechanisms may be occurring, possibly involving other aspects of the macrophage/microglia response.

Fingolimod (Gilenya) has demonstrated success in reducing the relapse rate as well as the rate of disability progression [116, 125]. It acts by modulating the sphingosine 1-phosphate receptors, thereby preventing lymphocyte recruitment, as these receptors are required for the exit of lymphocytes from secondary lymphoid organs to the CNS [126]. In addition, fingolimod induces an anti-inflammatory phenotype in activated macrophages. This is supported by the observation of decreased production of proinflammatory cytokines and free radicals by macrophages upon application of this drug [127].

Mitoxantrone (Novatrone) is a cytotoxic agent that functions through the intercalation of DNA [128]. Due to this broad mechanism of action, it affects many of the different types of immune cells, resulting in widespread immunosuppressive activity [129]. This immunosuppression results in the inhibition of monocyte and lymphocyte migration into the CNS [130]. Reductions in the proinflammatory cytokines, TNF-*α*, IL-2, and IFN-*γ*, have also been observed [131]. This reduction in monocyte activity may explain an observation made in which macrophage-mediated myelin degradation was inhibited [132].

Dimethyl fumarate (Tecfidera) is a recently approved first-line therapy for multiple sclerosis. It is an oral tablet thought to act by decreasing the expression of NF-*κ*B dependent genes [133, 134]. These genes regulate the expression of inflammatory cytokines, and as such, studies have reported decreased production of TNF-*α*, IL-1*β*, IL-6, and NO in microglia [135]. Adding to these effects, when dimethyl fumarate is administered to mice with EAE, a significant reduction in infiltrating macrophages is observed in the lesions [136]. As is the case with the aforementioned medications, there may be many modes of action that are still unknown. Nonetheless, involvement of macrophage/microglia seems to be consistent across the approved medications used for multiple sclerosis.

## 15. Conclusion

Macrophages/microglia are implicated in promoting demyelination; yet remyelination in MS lesions appears to require these cells as repair occurs in the presence of macrophages/microglia [137, 138]. From the research conducted on macrophages/microglia in models of CNS injury, including EAE, cuprizone-, and lysolecithin-induced demyelination, it is evident that a complex dichotomy exists in the roles of these cells in the demyelination/remyelination process. More detailed studies analyzing the spectrum of activities of macrophages/microglia at different time points in the focal demyelination models, as well as studies depleting and activating these cells at different temporal points will only serve to dissect the dichotomy presented to us. A thorough understanding of when macrophages/microglia are beneficial or detrimental in the demyelination/remyelination process will allow us to develop therapeutic interventions which take advantage of the respective characteristics, with the ultimate goal to enhance remyelination and suppress demyelination. In addition to the importance of the cuprizone and lysolecithin demyelination models in being critical tools for dissecting the dichotomy of macrophages/microglia, studies using EAE will serve to complement what we learn from the focal demyelinating models, altogether serving to further our understanding of the complex processes of de-/remyelination in injuries to the CNS.
